# Detection of Bronchial Neoplasia in Uranium Miners by Autofluorescence Endoscopy (SAFE-1000)

**DOI:** 10.1155/DTE.5.91

**Published:** 1999

**Authors:** T. Horvath, M. Horvathova, F. Salajka, B. Habanec, L. Foretova, J. Kana, H. Koukalova, P. Pafko, F. Wurst, E. Novotna, J. Pecina, V. Vagunda, R. Vrbacky, R. Talac, H. Coupková, Z. Pacovsky

**Affiliations:** 1 Masaryk Memorial Cancer Institute Zluty kopec 7 Brno CZ-65653 Czech Republic; 2 Clinic of Respiratory Medicine Masaryk University Hospital Bohunice Brno Czech Republic; 3 Department of Pathology Medical College Hospital of Johann Gregor Mendel Brno Czech Republic; 4 Department of Pneurnology Uranium Mines Hospital Pribram Czech Republic; 5 Third Clinic of surgery Charles University Hospital Motol Prague Czech Republic; 6 Department of Respiratory Medicine Hospital Decin Czech Republic

## Abstract

The increase in the detection rate for premalignant changes of bronchial epithelium was studied in 56 symptom-free volunteers from the risk group of Czech uranium miners (mean age 50.69 years, mean WLM 21.06 (1 Working Level Month is equal to the absorption of latent energy of 2.08 × 10^–5^ J/m^3^ in one month, i.e. 170 working hours)) by the additional employment of the System of Autofluorescence Endoscopy (SAFE-1000 Pentax) to conventional white-light bronchoscopy, comparing results with those of bronchial biopsy histopathology examination. Histopathology using hematoxylin and eosin staining confirmed intraepithelial neoplasias in 15 areas in 10 persons. White-light bronchoscopy sensitivity was 21.05%, and specificity 93.7% which an autofluorescence bronchoscopy sensitivity was 78.95% and specificity 81.89%.


*Diagnostic and Therapeutic Endoscopy*, Vol. 5, pp. 91–98
Reprints available directly from the publisher
Photocopying permitted by license only

© 1999 OPA (Overseas Publishers Association) N.V.
Published by license under
the Harwood Academic Publishers imprint,
part of The Gordon and Breach Publishing Group.
Printed in Singapore.

### Detection of Bronchial Neoplasia in Uranium Miners by Autofluorescence Endoscopy (SAFE-1000)*

T. HORVATH[a,†], M. HORVATHOVA[a], F. SALAJKA[b], B. HABANEC[c], L. FORETOVA[a], J. KANA[d], H. KOUKALOVA[a], P. PAFKO[e], F. WURST[f], E. NOVOTNA[a], J. PECINA[a], V. VAGUNDA[a], R. VRBACKY[a], R. TALAC[a], H. COUPKOVÁ[a] and Z. PACOVSKÝ[a]

[a]*Masaryk Memorial Cancer Institute, Zluty kopec 7, CZ-65653, Brno, Czech Republic, Europe;* [b]*Clinic of Respiratory Medicine, Masaryk University Hospital, Bohunice, Brno;* [c]*Department of Pathology, Medical College Hospital of Johann Gregor Mendel, Brno;* [d]*Department of Pneumology, Uranium Mines Hospital, Pribram;* [e]*Third Clinic of Surgery, Charles University Hospital Motol, Prague;* [f]*Department of Respiratory Medicine, Hospital Decin*

**The increase in the detection rate for premalignant changes of bronchial epithelium was studied in 56 symptom-free volunteers from the risk group of Czech uranium miners (mean age 50.69 years, mean WLM 21.06 (1 Working Level Month is equal to the absorption of latent energy of 2.08 × $10^{-5}$ J/m$^3$ in one month, i.e. 170 working hours)) by the additional employment of the System of Autofluorescence Endoscopy (SAFE-1000 Pentax) to conventional white-light bronchoscopy, comparing results with those of bronchial biopsy histopathology examination. Histopathology using hematoxylin and eosin staining confirmed intraepithelial neoplasias in 15 areas in 10 persons. White-light bronchoscopy sensitivity was 21.05%, and specificity 93.7% which an autofluorescence bronchoscopy sensitivity was 78.95%, and specificity 81.89%.**

*Keywords:* Autofluorescence, Bronchoscopy, Dysplasia, Intraepithelial neoplasia, Uranium miners

### INTRODUCTION

Lung cancer is the final stage of a multistep carcinogenic process [1]. The intraepithelial stage of neoplastic development typically lasts for a number of years before invasion occurs [2,3]. In uranium miners 68% of lung cancers are located in the central area of the bronchial tree [4].

Mining uranium ore is a high risk factor for lung cancer which can act after many years leaving the job in the mines. When the symptoms occur, the disease is usually advanced [5].

Lung cancer is recognized as an occupational disease in about 75 Czech uranium miners annually [6], but the real incidence is much higher and reaches some hundred cases per year. Epidemiologic studies

* PS-AFB98: Pilot study – Autofluorescence bronchoscopy 1998, SAFE-1000.

† Corresponding author. Tel.: ++420 5 4313 1111. Fax: ++420 5 4321 1169. E-mail: horvath@mou.cz.

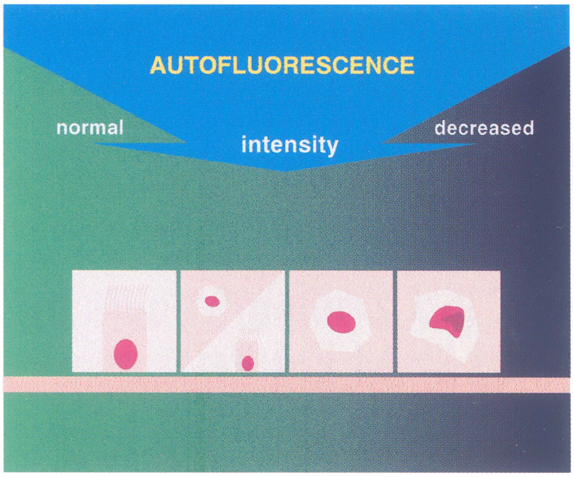

FIGURE 1 Autofluorescence – schema.

show the most endangered individuals are the miners who began work between 20 and 30 years ago [7,8].

The approach to lung cancer in this high risk group needs to focus attention on detection of premalignant and early malignant lesions and to try to prevent the occurrence of malignancies. Precancerous lesion and early lung cancers are difficult to detect with conventional white-light bronchoscopy (WLB) [9]. Woolner *et al.* noted that only 29% of all centrally located squamous cell carcinoma *in situ* were visible to an experienced bronchologist [10].

Kato *et al.* devised a simple fluorescence bronchoscope system with a conventional Xenon lamp excitation for detection of autofluorescence (Figure 1) without employing a laser [11,12].

### MATERIALS AND METHODS

In the prototype of the System of Autofluorescence Endoscopy (SAFE-1000, PENTAX, Tokyo, Japan) infrared light from a Xenon light source Pentax LX-750P is eliminated by a special infrared filter, and only blue light of 420–480 nm is delivered through an excitation filter and transmitted via a light guide. The emitted autofluorescence is transmitted via an image guide to a TV camera with a Hamamatsu ICCD Camera Controller C 3510, fluorescence filter which transmit only 520–600 nm light to an image intensifier which amplifies to the light. This core of the system is attached to the eyepiece of a standard Pentax bronchoscope (FB 18RX) and to the image monitor (Sony).

### Subjects

Seventy five subjects were examined, 56 persons of whom satisfied the conditions of the study: symptom-free volunteers from risk group of Czech uranium miners employed ten or more years in uranium mines with WLM evaluated cummulative radon exposure. They included smokers, ex-smokers and non-smokers.

### Clinical Protocol

Conventional fiberoptic bronchoscopy was performed under local anesthesia. First WLB examination was done, then autofluorescence bronchoscopy (AFB) by the SAFE-1000 device. Areas suspected of premalignant changes were studied with alternating white and blue light and photographs were taken. Both investigations were recorded on videotape. Biopsy specimens for histopathology were taken from all suspected areas on WLB and AFB. Then random biopsies were carried out in visually normal areas using both techniques from right carina 2 or 1 (RC2 and RC1) and left carina 1 or 2 (LC1 and LC2), and other places as needed. Biopsy specimens underwent standard staining hematoxylin and eosin (HE). They were examined by an experienced pathologist.

All subjects gave informed consent. The approval for the study was given by the Ministry of Health Care of the Czech Republic and Czech Medical Chamber.

### RESULTS

### Data Analyses

The aim of the study was to evaluate the presence of premalignant changes of bronchial mucosa in Czech uranium miners and the sensitivity of the SAFE-1000 in conjunction with conventional WLB using bronchial biopsy histopathology for objective of comparison of visual findings of both endoscopy methods. The 157 biopsies performed in 56 subjects were divided into the 3 groups:

Group 1 consisted 108 random biopsies from apparently normal areas on autofluorescence and WLB, of this 104 specimens showed normal or mild inflammatory mucosal finding, and 4 specimens were found to show mild intraepithelial premalignant changes of bronchial mucosa (1 basal cell hyperplasia, 1 mature squamous metaplasia, 2 mild dysplasias).

Group 2 included 11 biopsies with alternating normal respiratory epithelium and squamous metaplasia without atypia. They were not included in AFB sensitivity estimation.

Group 3 consisted 38 specimens from areas with decreased autofluorescence intensity. Histopathology examinations confirmed 15 areas with intraepithelial premalignant changes (i.e. true positive AFB findings), and 23 areas exhibited no premalignancy (i.e. false positive AFB findings). False positive AFB findings from the histopathologic point of view consisted 18 specimens which were found to be normal and 5 biopsies which were found to show inflammation.

Groups 1 and 3, totally 146 specimens, were included for estimation of AFB sensitivity in 56 persons aged 39–72 (mean 50.69), mean years entered to risk 22.48 years, at risk exposure 14.55 years, after exposure 12.20 years, mean WLM 21.06, among them these were 25 smokers (44.64%), 3 ex-smokers (5.36%) and 28 never-smokers (50%). Significant decrease of autofluorescence intensity was seen in 38 areas (Table I). Histopathology confirmed premalignant changes in 15 areas, 12 were found to be dysplasia, including 5 with moderate dysplasia (Fig. 2) and 7 with mild dysplasia (Fig. 3), 2 were found to have metaplasia and there was 1 basal cell hyperplasia. Another 23 areas had no evidence of premalignancy (Table II). WLB showed pathological findings in 12 areas in 10 persons (Table III).

TABLE I AFB and biopsy results

| | Histopathology intraepithelial neoplasia | |
|---|---|---|
| | + | – |
| AFB | | |
| + | 15 | 23 |
| – | 4 | 104 |
| | 19 | 127 |

The 10 subjects included 6 smokers, 1 ex-smoker and 3 non-smokers. Histopathology confirmed intraepithelial neoplasia in 4 areas (3 subjects), other 8 areas were found premalignancy-free (Table IV).

The group of 13 persons (19 areas) with evidence of intraepithelial neoplasia (true positive and false negative findings) included 3 non-smokers, 1 ex-smoker and 9 smokers.

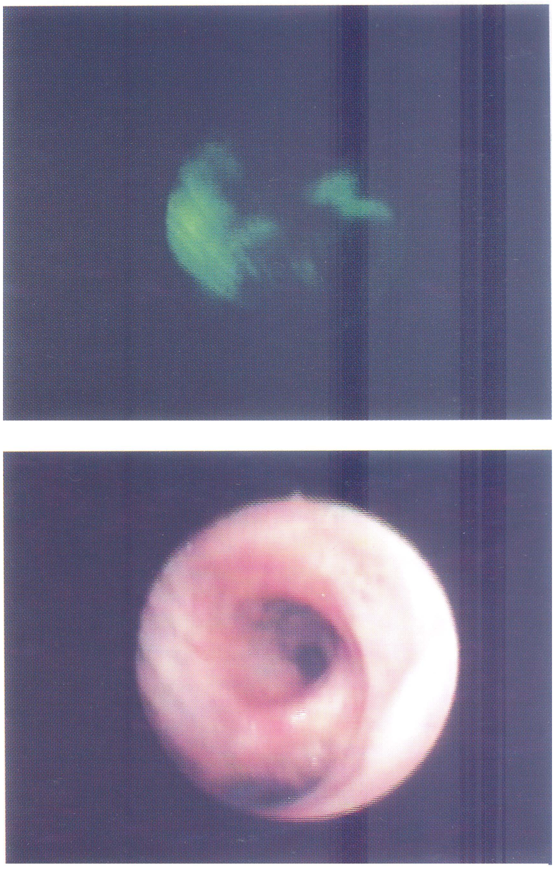

FIGURE 2 Moderate dysplasia – photo.

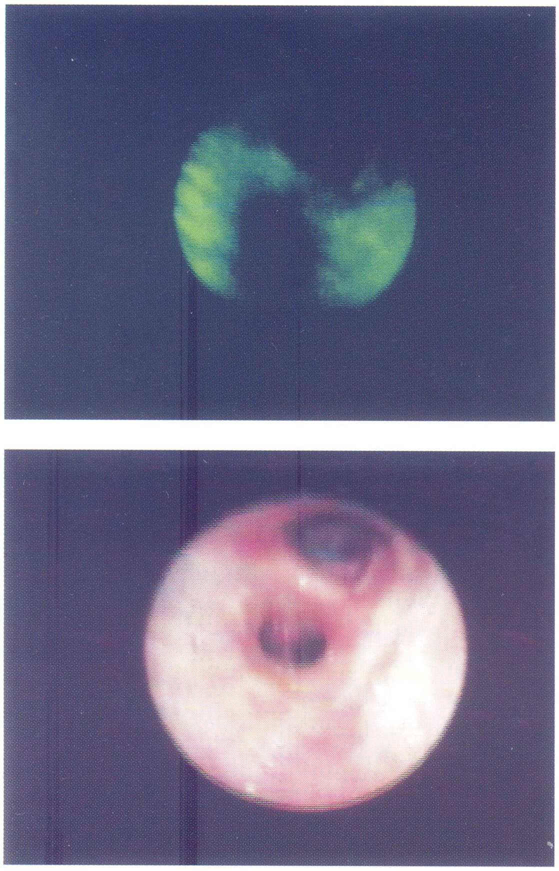

FIGURE 3 Mild dysplasia – photo.

The examinations had no complications. The average time of the procedure was 21 minutes (range 7–34 min). The sensitivity analysis of (1) WLB sensitivity 21.05%, specificity 93.7%, false positive rate 6.3%, false negative rate 78.95%, predictive value of positive test 33.3%, predictive value of negative test 88.81%, accuracy 84.25% and (2) AFB sensitivity 78.95%, specificity 81.89%, false positive rate 18.11%, false negative rate 21.05%, predictive value of positive test 39.47%, predictive value of negative test 96.3%, accuracy 81.51% were obtained.

### DISCUSSION

In order to control and reduce mortality prevention and early detection are necessary. Autofluorescence imaging facilitates the study of the natural history of

TABLE III WLB and biopsy results

| | Histopathology intraepitheilal neoplasia + | Histopathology intraepitheilal neoplasia – |
|---|---|---|
| WLB | | |
| + | 4 | 8 |
| – | 15 | 119 |
| | 19 | 127 |

TABLE II AFB sensitivity

| AFB | Number of biopsies | Histopathology ($n = 56$) |
|---|---|---|
| *Included* | | |
| Positive | | |
| True | 15 | 5 moderate dysplasias<br>7 mild dysplasias<br>2 metaplasias<br>1 basal cell hyperplasia |
| False | 23 | 5 inflamed<br>18 normal |
| Negative | | |
| True | 104 | Normal and/or mild inflammation |
| False | 4 | 2 mild dysplasias<br>1 metaplasia<br>1 basal cell hyperplasia |
| *Non-included* | | |
| Positive | 1 | Normal/metaplasia |
| Negative | 10 | Normal/metaplasia |
| Total | 157 | |

TABLE IV WLB sensitivity

| WLB | Number of biopsies | Histopathology ($n = 56$) |
|---|---|---|
| *Included* | | |
| Positive | | |
| True | 4 | 2 mild dysplasias<br>1 metaplasia<br>1 basal cell hyperplasia |
| False | 8 | 4 inflammations<br>1 cicatricial formation<br>2 normal<br>1 anthracotic pigmentation |
| Negative | | |
| True | 119 | Normal, inflammation, other |
| False | 15 | 5 moderate dysplasias<br>7 mild dysplasias<br>2 metaplasias<br>1 basal cell hyperplasia |
| *Non-included* | | |
| Negative | 11 | Normal/metaplasia |
| Total | 157 | |

TABLE V Sensitivity and specificity AFB/WLB

| | White-light bronchoscopy (%) | Autofluorescence bronchoscopy (%) |
|---|---|---|
| Sensitivity | 21.05 | 78.95 |
| Specificity | 93.70 | 81.89 |

lung cancer, provides a means of establishing more precise intermediate end points in investigations of efficacy of chemopreventive drug and has important implications in the treatment of patients with lung cancer.

This pilot study proved the sensitivity of the prototype autofluorescence system for lower grade atypia of bronchial mucosa. The difficulties at the beginning of the learning curve have gradually disappeared. The AFB has 3.75-times higher sensitivity than WLB but about 12% lower specificity (Table V).

From the analysis of causes of false positivity (Fig. 4 and Table VI) the groups I, II and IV with normal histopathology, group III with pathological findings and group V revealed various causes such as anatomically related shadows (SAC – e.g. Fig. 5), by motility related abnormality of decreased autofluorescence intensity (MOD), adherent mucus

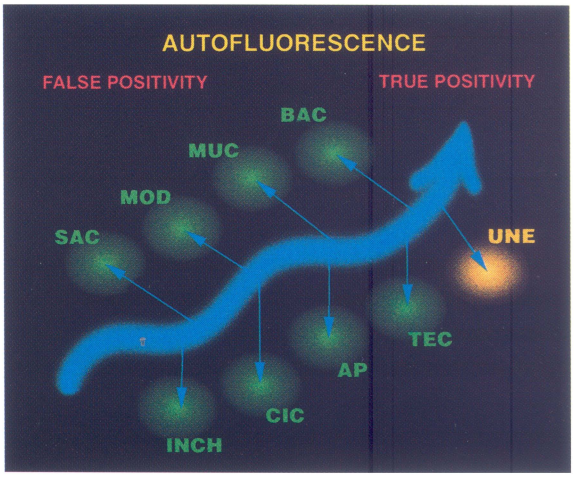

FIGURE 4 False positivity – schema.

(MUC – Fig. 6), biopsy accuracy (BAC) technique, light intensity, effects of distance, etc. (TEC), anthracotic pigmentation (AP). They emphasize the necessity for improvement of AFB sensitivity through training, with regard to inflammatory changes (INCH) and cicatricial formation (CIC) from group II seem to be relative common causes of false positive results of AFB. Unexplained causes (UNE) remain as a challenge for the future programs of this technique.

TABLE VI Causes of false positivity of AFB

| | Number | Remark |
|---|---|---|
| 1. *Anatomy and physiology* | | |
| SAC Shading due to anatomically configuration | >10 | WLB |
| MOD Decreased autofluorescence affected by motility | >4 | TEC |
| MUC Mucus | >6 | WLB |
| II. *Examinator and equipment* | | |
| BAC Biopsy accuracy | >1 | ! |
| TEC Technique/equipment | >2 | MOD |
| III. *Pathology* | | |
| INCH Inflammatory changes | 5 | WLB |
| CIC Cicatricial formation | 3 | History |
| AP Anthracotic pigmentation | >2 | WLB |
| IV. | | |
| UNE Unexplained | 3 | WLB |
| V. | | |
| Combinations (I–IV) | X | Frequently |

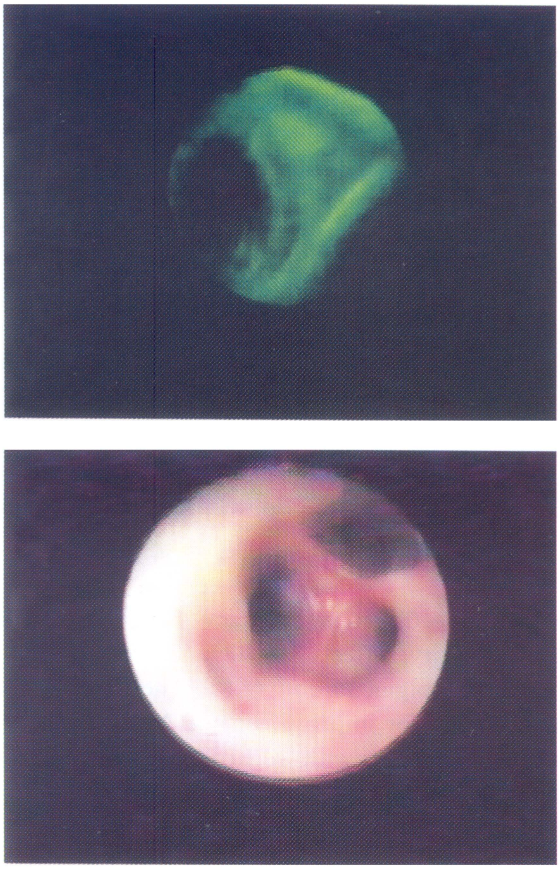

FIGURE 5 False positivity – Shading due to anatomically configuration (SAC).

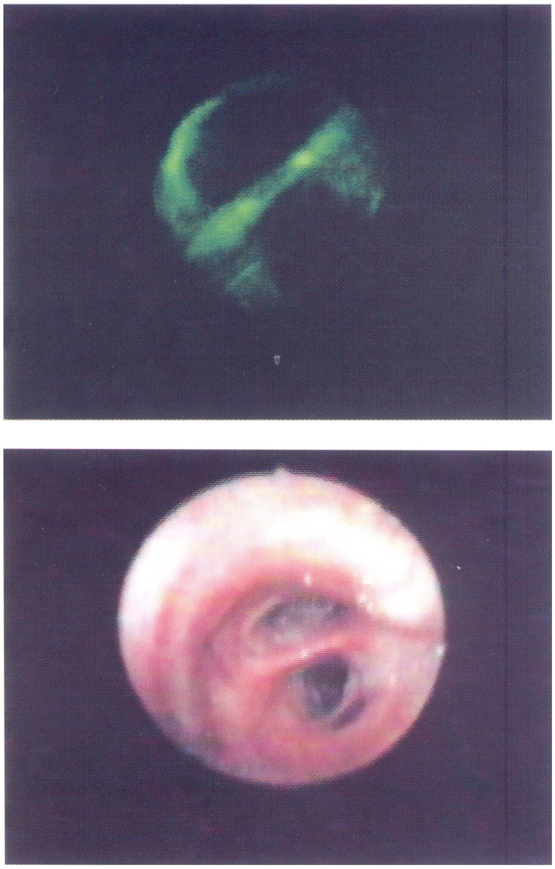

FIGURE 6 False positivity – Mucus (MUC).

This pilot study raises the question of borderline autofluorescence diagnosis of emerging atypia. Accurate evaluation was difficult in 11 specimens. They were classified as checkered findings according to histopathology, with alternating normal respiratory epithelium and squamous metaplasia without atypia. Ten specimens were located in areas of normal autofluorescence intensity i.e. visually negative AFB findings. The other specimen of this type came from an area with decreased autofluorescence intensity i.e. visually positive AFB findings. The 11 specimens were not included in the evaluation of AFB sensitivity. Further examinations of specimens and follow-up investigation of the involved areas are necessary.

All false negative biopsies (Fig. 7) were found to be areas of low grade intraepithelial premalignancy. Thin epithelial layers could explain the false negativity of 3 specimens, 1 with basal cell hyperplasia, 1 with mature squamous metaplasia, and 1 with mild dysplasia. False negativity of the second biopsy with mild dysplasia could have been caused by low grade atypia, by the biological properties of the tissue, or by technical reasons.

The detection of basal cell hyperplasia based on autofluorescence requires consideration of some of the causes of false positivity and optically non-significant presence of basal cell hyperplasia in the epithelium, the biological properties of the bronchial tissue and also the technical characteristics of the equipment.

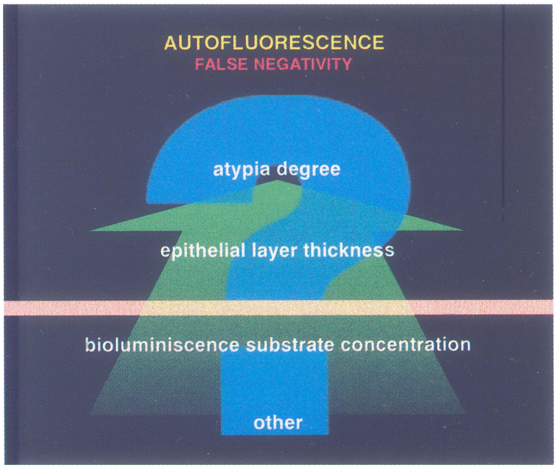

FIGURE 7 False negativity – schema.

The development of methods for detection and treatment of intraepithelial lesions within the bronchial tree is promising both from surgical and medical points of view [11–16]. Autofluorescence technology, endobronchial sonography, ultrathin bronchoscopy, chemoprevention, photodynamic therapy and electrocautery offer challenges to work together.

*Acknowledgements*

The authors express deepest thanks to Prof. Harubumi Kato and his team from Tokyo Medical College for outstanding support, to Pentax company for excellent technical support, to Prof. J. Patrick Barron for his kind review of the manuscript, to many others who found the courage to help start the project, but stay incognito in the background.

### *References*

[1] Auerbach, O., Stout, A.P., Hammond, E.C. *et al.* Changes in bronchial Epithelium in relation to cigarette smoking and in relation to lung cancer. *N. Eng. J. Med.* 1961; **265**: 253–267.
[2] Woodruf, M.F., Tumor clonality and its biological significance. *Adv. Cancer Res.* 1988; **50**: 197–229.
[3] Saccomanno, G., Archer, V.E., Auerbach, O. *et al.* A Comparison between the localisation of lung as reflected in exfoliated cells. *Cancer* 1974; **33**: 256–270.
[4] Saccomanno, G., Auerbach, O., Kuschner, M. *et al.* A comparison between the localisation of lung tumors in uranium miners and nonminers from 1947 to 1991. *Cancer* 1996; **77**: 1278–1283.
[5] Prochazka, F. *Occupational Diseases* (in Czech). Snajdr, Kladno, 1933.
[6] Vejlupkova, J. and Urban, P. Overview of occupational diseases in Czech Republic in year 1994. *Prac. lek.* 1995; **47**: 99–104.
[7] Ševc, J., Tomášek, L., Plaček, V. Risk of malignant lung tumours as a result of inhalation of radon daughter products. *Cs. hyg.* 1991; **36**: 3–13.
[8] Tomasek, L., Darby, S.C., Fearn, T. *et al.* Patterns of lung cancer mortality among uranium miners in West Bohemia with varying rates of exposure to radon and its progeny. *Radiation Research* 1994; **137**: 251–261.
[9] Horvath, T., Horvathova, M., Hruska, P. *et al.* State of bronchial mucosa in Czech uranium miners. A pilot study. *Eur. Respir. J.* 1997; **10**: Supplement 25, 114s, P0758.
[10] Woolner, L.B., Fontana, R.S., Cortese, D.A. *et al.* Roentgenographically occult lung cancer. Pathologic findings and frequency of multicentricity during a 10 year period. *Mayo. Clin. Proc.* 1984; **59**: 453–466.

[11] Kato, H., Okunaka, T., Ikeda, N. and Konaka, C. Application of Simple Imaging Technique for Fluorescence Bronchoscope. Preliminary Report. *Diagnostic Therapeutic Endoscopy* 1994; **1**: 75–79.

[12] Kato, H., Konaka, C., Okunaka, T., Furukawa, K. and Ikeda, N. Early diagnosis of central type lung cancer with bronchoscope. *Medicina Thoracalis* 1997; **50**: 131–143.

[13] Hayata, Y., Kato, H., Konaka, C. and Okunaka, T. Photodynamic therapy (PDT) in early stage lung cancer. *Lung Cancer* 1993; **9**: 287–294.

[14] Lam, S., MacAulay, C., Hung, J. and Palcic, B. Detection of dysplasia and carcinoma in situ with a lung imaging fluorescence endoscope device. *J. Thorac. Cardiovasc. Surg.* 1993; **105**: 1035–1040.

[15] Boone, C.W., Kelloff, G.J. and Steele, V.E. Natural history of intraepithelial neoplasia in humans with implications for cancer chemoprevention strategy. *Cancer Res.* 1992; **52**: 1651–1659.

[16] Sutedja, T.G. *et al.* Endobronchial Electrocautery is an excellent alternative for Nd-YAG laser to treat airway tumors. *J. Bronchol.* 1997; **4**: 101–105.

